# Dr. Farre, on the Morbid Anatomy of the Liver

**Published:** 1816-05

**Authors:** 


### Dr. Farre, on the Morbid Anatomy of the Liver.

(Continued from page 348.)

The *Second Part* comprehends the *varieties* of the Tubera Diffusa. They are very numerous. Dr. Farre selects for delineation four of them, most strikingly distinguished by peculiarities of anatomical character, and elucidates them by cases; but he does not venture to assign them a name.

II. 1. In this first variety, the anatomical peculiarities of the two species of Tuber are combined; and hence it serves to connect them.

"*Character.* Tubera, affecting different textures, viz. those of the liver and stomach; elevated at the surfaces of the organs, but not uniform in their figure; some rising with a regular swell into a round form, others acquiring a margin by being gradually depressed towards the centre; forming tumors without cysts, almost pulpy in their consistence, cellular in their structure, and containing an opaque white fluid."

*Symptoms.* These are proportioned to the development of the tumors in the respective organs. The symptoms attendant on the T. circumscripta, with the addition of those signs which result from the affection of the stomach, will serve to distinguish the genus and species, but not the variety. Our limits will allow us to trace but a mere outline of the history and dissection of the case which illustrates this variety. The subject, a female, married, temperate, commonly healthy, had formerly suffered attacks of jaundice and pleurisy. Seized, at the close of 1813, with dyspepsia, burning in the stomach, and pain extending to the shoulders. Relieved, for a time, by weaning her child (the sixth). Symptoms returned; alvine constipation. Violent palpitations, with syncope, not affecting the pulse; dyspnœa on ascending stairs; occasional pain in the left side, and dry cough, succeeded. Cough ceased; but emaciation began. In April, 1814, enlargement of the liver was detected. The character of the disease was announced by its rapid growth. In six days after, tubera were distinctly felt on the liver. The patient "compared them to eggs, and said she could distinguish the rise of the tumours by the exquisite pain and tenderness which attended their rapid growth." She could only lie on her back. Emaciation went on. In the beginning of May, the liver had much increased; strength declining; slight peritoneal effusion; urine scanty, turbid, pink-coloured; pulse 96. Dropsy and wasting increased. Difficulty of motion, and pain formerly attendant on vomiting, much lessened, probably by the interposition of fluid between the peritoneal surfaces. Diarrhœa manifested itself; tongue clean, but dry; thirst urgent. Vomiting now productive of relief from pain and sense of burning, consequent on ingestion of food; the ejected fluids coffee-coloured. Great pain between the shoulders. Legs became œdematous. Fæces now white. Continual heat and pain in the stomach; pains in the hips; sense of suffocation; distress in deglutition, and occasional hiccough, terminated by death about the middle of May.—On dissection, the liver was seen almost reaching to the pelvis: left lobe nearly destroyed by Tubera, which unequally distended its surfaces, but left its edge thin and flowing. Texture of the right lobe not so universally implicated.

"The structure of the tubera was cellular, not encysted, and the cells contained the usual cream-like fluid. The external surfaces of most of the tubera were uniformly elevated, or round; but some, on the contrary, were depressed in the centre, or indented, as in the Tubera circumscripta."

The gall bladder contained biliary concretions; the cystic duct was impervious. The lymphatic glands between the liver and stomach were enlarged. A large flat or slightly concave tuber grew from the mucous coat of the stomach near the pylorus. Some fluid was effused into the peritonæum. There was a trifling effusion of blood beneath the adherent portion of the pericardium. A lymphatic gland near the trachea was altered in structure, but not tuberous. This variety is depicted.

II. 2. "*Character.* Tubera affecting different textures, elevated at the surfaces of the affected organs, encysted, or having distinct cells, formed by the growth of a fungus, which separates in flakes, and is composed of a fine reticular texture, containing an opaque white fluid."

We may be enabled by the *symptoms* to discriminate the species, but not this variety. The following is Dr. Farre's illustrative case, in an abridged form:—A female, aged 48, small in figure, and temperate, had complained, for two or three years, of epigastric pains. During the last twelve months, frequent vomiting; bowels regular. More recently attacked by ascites and jaundice; latter disappeared a month before death. Medicine only aggravated the gastric pain, expressed by constant friction of the region of the stomach with her hand. Respiration little affected; pulse small and quick; debility great.—Dissection. Liver not remarkably enlarged; externally irregular from numerous tubera; internally, filled with them. Some of the tubera had distinct cells, or even cysts, which contained a white substance of the consistence of cheese, capable of being separated in flakes. From the surfaces of the tubera, after a fresh section, a white fluid, as thick as cream, could be scraped. A portion of the tumour, after many days' maceration, presented a fine reticular texture as the structure of the tubera, in which that fluid was contained. An irregular tuber grew from the internal coat of the stomach, near the pylorus. The peritonæum contained six pints of fluid. This variety is delineated, Plate II. fig. 2.*

II. 3. " *Character.* Tumors rising with a regular swell from the surfaces of the affected parts, and yielding to the touch; composed of a very delicate reticular texture, pulpy in its consistence, varying in its colour, even in the same subject, charged with an opake fluid, and growing from cysts or cells."

*Symptoms.* This variety can only be distinguished from the preceding by the pulpy consistence of the tumors, which yield a sensation somewhat resembling that of deep-seated fluid. In every instance yet observed by Dr. Farre, this variety of tuber, when occupying the liver, was secondary: the primary arising in distant organs or in cellular membrane. The fungus in the former was always attached to the *interior* of cysts or cells; in the latter, it sometimes grew from the *exterior* surfaces.—The preced-

* This, Dr. Farre was formerly led to consider, and to mark, as the *first* variety. Subsequent observation has induced him to class it as the *second.*

ing varieties of tuber are confined principally, if not exclusively, to the adult organs. From the ravages of this third, no age, no structure, cartilage perhaps excepted, is exempt. The internal membranes of the eye, the female breast, the testicle, often sustain its first attacks. In the latter, it constitutes the disease known in the London schools by the name of the *pulpy testicle.*—The following history, first of the three presented by Dr. Farre, very clearly elucidates this variety.

A tumor appeared in the left umbilical region of a child, soon after birth, and gradually increased. At the age of three months, vomiting came on. The swelling, as it enlarged, seemed to consist of distinct tumors. Continual draining of blood, with discharge of clots from the bladder. The tumor pointed in several places; the child sank rapidly, and died at the age of five months. The tumor had been, at first, considered as an abscess. Mercury had been internally and externally employed, with fomentations.

Dissection. " Abdomen. The intestines appeared in the epigastric region, except portions of the colon and rectum, which were pressed forward to the anterior parietes of the abdomen, by a very large tumor which originated behind the peritonæum, and enveloped the left kidney. The tumor was encysted, and its pulpy contents were compared to putrid brain. At the surface of the liver appeared swellings, which, both to the touch and eye, seemed to be in a state of complete suppuration; but this appearance was deceptive, for an incision being made through this organ, its substance was manifestly pervaded by secondary tumors of the same structure as the large or primary tumor. Numerous cysts, varying much in size, and most intimately connected with the liver, were filled with a fungus which broke down under very slight pressure. This substance was of a reddish-brown colour, but under maceration it soon became white; and, by expanding itself, more perfectly manifested its fungous character. When macerated for a few days, on being slightly agitated in water, its filaments were readily separated from their cysts, the internal surfaces of which were left uneven with small membranous laminæ."

The coats of the stomach were corroded by the gastric juice; other abdominal organs sound.

" Thorax. The lungs were covered with numerous prominent tubera, of a smaller size than those in the liver, containing the same morbid structure; but maceration proved, that it adhered more intimately to the substance of the lungs than it did to the liver."

The heart and brain were sound. About an ounce of fluid was collected between the dura and pia mater.—

Fig. 1, plate III. represents the external character of this variety of tuber. We must not pause to notice the two succeeding cases, farther illustrative of it; but pass on to consider the fourth and last variety presented by Dr. Farre.

II. 4. " *Character.* Tumors elevated at the surfaces of the liver, and inclining to a round form; pulpy in their consistence, being charged with a thick and opaque fluid; variegated in their colour, chiefly white mingled with red, the former prevailing in their incipient, the latter in their advanced stages; composed of a very vascular and reticular texture, attached either to distinct pouches, or to the substance of the liver; and *so unlimited and rapid in its growth, as to burst or destroy the peritoneal tunic of this organ, and to protrude in the form of a bleeding fungus.*" *

This variety cannot be distinguished from the last, by the symptoms, during life. We shall concisely present the only example by which it is here illustrated.—A man, aged 41, large, vigorous, athletic, intemperate, subject to indigestion, suffered, in November 1812, from pain in the left hip and iliac region. Epigastric pains succeeded. The liver was found enlarged and tuberous. Debility; bad appetite; pain above the umbilicus, particularly in the regions of the stomach and liver, tense and painful on examination, were observed towards the close of November. The hepatic enlargement and inequalities of surface became more distinctly marked as emaciation proceeded. The organ extended almost to the umbilicus, and pressure on various parts was unequally painful. Countenance sallow, without jaundice; urine turbid, sedimentous; stools variable, but not indicating a very deficient biliary secretion. Pulse 80, more frequent towards the close of life. Little dyspnœa; no cough. Vomiting, singultus, and peritoneal effusion, preceded death, which happened on the 27th of December. Body examined on the 30th. Opium only had afforded temporary relief from severe suffering.

By pressure of the swollen abdomen, the enlarged liver, and two tubera on its surface, could be distinctly traced.

" On dividing the parietes, many quarts of serum, deeply coloured with red particles, escaped, and numerous filaments of crassamentum were found adhering to the intestines. A consider-

* The *fungus hæmatodes* of Mr. Hey. Dr. Farre complains, that the term has been indiscriminately extended to many varieties of tuber, " notwithstanding the absence of even the very fact which authorized the name."

able portion of the liver appeared below the hypochondria, and occupied the whole of the epigastric and a part of the umbilical regions. Numerous tubera were seen on the convex and concave surfaces of both lobes. They had a motley appearance, and rose with a gradual swell from the liver. Their contents were pulpy, of a whitish colour mingled with red, and, when compressed, a cream-like fluid, tinged with blood, rose in abundance from their reticular texture. In two distinct parts of the right lobe, its peritoneal tunic had been ruptured by soft fungi, of a red colour, from which the sanies had issued. Some of the tubera were distinct, the fungous structure being attached to cells, and the intervening portions retaining the character of liver; but many of the tumors coalesced, and the boundaries between the natural and morbid structures were indistinct. The gall-bladder contained a very little bile of a bad quality. The parts about Glisson's capsule were so united into one mass, as to prevent any dissection of the biliary ducts and vessels. The stomach, being thrust much out of its natural position by the enlarged liver, and by the clusters of tubera which occupied its lesser curvature, and surrounded its pyloric extremity, appeared unusually low on the left side. Its tubera were still more pulpy than those of the liver; and their fungous structure, of a very red colour, was either contained in a large cyst, or grew from numerous small and very distinct pouches. The mucous coat of this organ was much thickened, and its surface was irregular. Here and there a small cyst appeared in it, filled with the fungous structure. This coat was so much thickened as to narrow the pylorus, and so pulpy on its surface at that extremity as to present an appearance resembling ulceration. Numerous lymphatic glands in the course of the aorta were converted into tubera."

The other internal organs were sound. The external and internal appearances of the tubera affecting the liver of this subject are exquisitely depicted, plate IV. fig. 2.

The two varieties, last described, are intimately related. They pass into each other. The medullary appearance, though common, does not invariably exist, and is peculiar to neither. Much obscurity prevails with respect to the mode in which the whole genus of tubera is produced. The third and fourth varieties of the second species afford, in Dr. Farre's opinion, the best opportunities of ascertaining it; but he has, at present, little to communicate on the subject. A liver, affected with the third variety of T. diffusa, was injected. The vessels of the cysts were filled; but the injection did not colour the fungus attached to them. Many such experiments must be made, ere the belief in this distinct line of separation between the morbid and natural structures can be implicitly admitted. The excessive hæmorrhages, which take place from the fungus in the fourth variety, seem to contradict this inference. Maceration he recommends as the best means of developing these morbid textures.

Tubera, it is lastly observed, increase by contiguity, and spread through the different textures by absorption, or a certain condition of the system not yet ascertained. To this latter their origin must be referred, whenever appearing successively out of the track of absorption.

Thus far our examination has been purely analytical. The few remarks which we have to make, will be reserved for our review of the third part of this elegant and masterly production. We are daily expecting its appearance, and not without impatience. There is a class of medical practitioners among us, who, at the very name of "liver disease," almost instinctively grasp the calomel bottle, and denounce against the shade of a "bilious symptom," however light and fugitive, salivation in all its terrors. To these we would beg leave to address a parting admonition. If the heresies and depredations of the mercurial sect will yield to any remedy short of "fire and faggot," the present work of Dr. Farre is surely calculated to annihilate or repress them. All such men, if such perchance there may be among our readers, we earnestly exhort not to rest satisfied with the outline here rudely traced, but to study, and glean conviction from, the pages of the original; not to content themselves with the spare and stagnant potion which we can only offer, but to allay the feverish heats of the unhappy mania which infests them, and wash away the load of noxious crudities by which they are oppressed with the "waters of the living stream."

